# The focal hepatic hot spot sign with lung cancer in computed tomography

**DOI:** 10.1002/rcr2.671

**Published:** 2020-10-07

**Authors:** Van Trung Hoang, Nhu Quynh Vo, Cong Thao Trinh, Hoang Quan Nguyen, Vichit Chansomphou, Trong Binh Le

**Affiliations:** ^1^ Department of Radiology Thien Hanh Hospital Buon Ma Thuot Vietnam; ^2^ Department of Radiology Hue University of Medicine and Pharmacy Hue Vietnam; ^3^ Department of Radiology Hue Central Hospital Hue Vietnam; ^4^ Department of Radiology Da Nang Oncology Hospital Da Nang Vietnam; ^5^ Department of Radiology Savannakhet Medical‐Diagnostic Center Kaysone Phomvihane Lao People's Democratic Republic

**Keywords:** Hot spot sign, lung cancer, superior vena cava obstruction

## Abstract

The focal hepatic hot spot sign appears as an area of intense focal wedge‐shaped enhancement of the quadrate lobe (segment IVa) of the liver in the arterial and venous phase. If this sign appears on enhanced computed tomography of the abdomen, obstruction of thoracic central venous must be considered, especially when clinical symptoms are unclear.

## Graphical abstract

The focal hepatic hot spot sign appears as an area of intense focal wedge‐shaped enhancement of the liver in the computed tomography images. We present this sign in a 60‐year‐old man with superior vena cava obstruction caused by lung cancer.

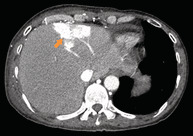

## Clinical Image

**Figure 1:**
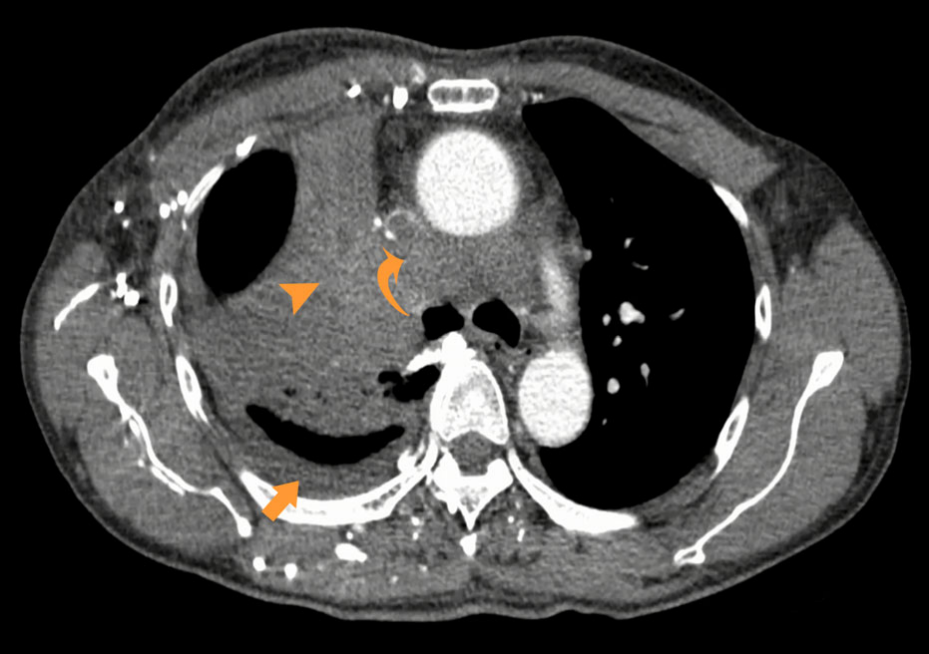
Axial contrast‐enhanced computed tomography (CT) of the chest shows the invasive right lung tumour (arrowhead) causing obstruction of superior vena cava (SVC; curved arrow). Note the presence of pleural effusion (arrow).

**Figure 2:**
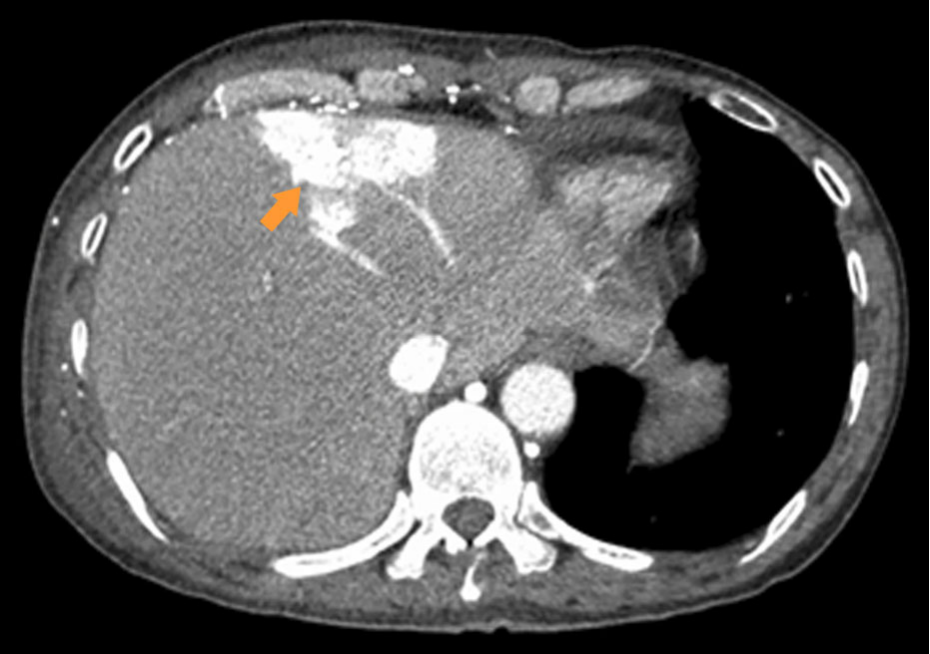
The focal hepatic hot spot sign on the axial contrast‐enhanced computed tomography (CT) image in the arterial phase. It manifests as an intense wedge‐shaped homogeneous enhancement area of size 4 × 6 cm in segment IV of the liver (arrow).

A 60‐year‐old man was hospitalized to continue chemotherapy for non‐small cell lung cancer diagnosed three months earlier (stage IVB right lung carcinoma, metastatic to the brain and lymph nodes, and superior vena cava (SVC) thrombosis). After being diagnosed, he was treated with docetaxel chemotherapy. This time, he underwent computed tomography (CT) of the chest and abdomen to re‐evaluate the invasion and progression of the lung tumour. CT of the chest indicated an invasive right lung tumour that caused an obstruction of the SVC with the dilated internal thoracic vein (Fig. 1). CT of the abdomen revealed an intense wedge‐shaped homogeneous enhancement area in the quadrate lobe of the liver (Fig. 2). The focal hepatic hot spot sign was first described in 1983 by Ishikawa. The sign can be observed on technetium 99m (99mTc) sulphur colloid scans or on contrast material enhanced CT scans. This abnormal enhancement is due to portosystemic venous shunting between the SVC and portal vein. The hot spot is created by areas of focally increased blood flow that result from this shunting [1]. This sign has been reported in Budd–Chiari syndrome, the causes of SVC syndrome (neoplasms of the thorax as lung carcinoma and lymphoma, Vasculo‐Behcet's disease, fibrosing mediastinitis, and luetic aneurysm), and masses of the liver (abscess, haemangioma, focal nodular hyperplasia, and hepatocellular carcinoma) [2].

### Disclosure Statement

Appropriate written informed consent was obtained for publication of this case report and accompanying images.
